# High‐Resolution Mapping of Discharge Product in Li─O_2_ Batteries

**DOI:** 10.1002/smtd.70699

**Published:** 2026-05-07

**Authors:** Laurence F. Brazel, Margherita Martini, Eric Maire, Arnaud Demortière, Michael De Volder, Clare P. Grey, Israel Temprano

**Affiliations:** ^1^ Yusuf Hamied Department of Chemistry University of Cambridge Cambridge UK; ^2^ INSA de Lyon CNRS MATEIS UMR5510 Université De Lyon Villeurbanne France; ^3^ Laboratoire de Réactivité et Chimie des Solides (LRCS) CNRS‐UPJV UMR 7314 Hub de l'Energie Amiens France; ^4^ FR CNRS 3459 Réseau Français sur le Stockage Electrochimique de l'Energie (RS2E) Amiens France; ^5^ FR CNRS 3104 ALISTORE‐European Research Institute Amiens France; ^6^ Institute for Manufacturing Department of Engineering University of Cambridge Cambridge UK; ^7^ CICA – Interdisciplinary Center For Chemistry and Biology University of A Coruña A Coruña Spain

**Keywords:** discharge product distribution, Li─O_2_ batteries, scanning electron microscopy, X‐ray computed tomography, X‐ray micro‐analysis

## Abstract

Lithium‐oxygen (Li─O_2_) batteries have the highest theoretical specific energy of any chemical battery (∼3500 Wh kg^−1^). However, their practical capacity is usually lower than theoretically expected, particularly at higher rates of discharge. Air electrode surface passivation and slow O_2_ mass transport through the porous electrode structure are the primary limitations on Li─O_2_ discharge capacity. To determine which of these factors is most significant, it is necessary to spatially determine the relative utilisation of different portions of the electrode. Here, we demonstrate how cross‐sectional scanning electron microscopy coupled with energy‐dispersive X‐ray spectroscopy (SEM‐EDS) can be used to semi‐quantitatively map the discharge product distribution within Li─O_2_ air electrodes. The distribution shows increased discharge product accumulation at the O_2_‐side of the electrode, confirming that pore blockage and oxygen starvation are key factors leading to under‐utilisation of the air electrode in Li─O_2_ cells. Simulated Li─O_2_ battery results align with the characterised discharge product distribution, lending validation to the model. Lab‐based X‐ray nano‐computed tomography corroborates the SEM‐EDS data to ensure that sample preparation had minimal effect on the measured distribution. Collectively, these techniques can be used to determine the cause of performance changes due to different discharge conditions, alternative electrolyte compositions, or electrode structure.

## Graphical abstract

Li─O_2_ batteries promise exceptional energy density, but have several factors limiting performance, such as poor rate performance. To understand rate limitations, scanning electron microscopy with energy dispersive X‐ray spectroscopy and X‐ray nano‐computed tomography were employed to map the distribution of discharge products in discharged air electrodes, and identify O_2_ mass transport as the primary rate limitation under the conditions tested.

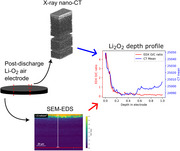

## 1 Introduction

There is a pressing need to reduce global reliance on fossil fuels as our primary energy storage medium to minimise greenhouse gas emissions and thus mitigate the impacts of climate change. Novel energy storage solutions are required to fully decarbonise our society across diverse sectors (e.g., electrical grids, transport, heating) [1]. Lithium‐ion batteries have become ubiquitous in recent years, being key components in the electrification of the transport industry [2], as well as being widely used for stationary storage [3]. However, they have a limited specific energy, typically around 300–400 Wh kg^−1^ [4], and limitations on transition metal supply may hinder their full proliferation [5]. Lithium‐oxygen (Li─O_2_) batteries could overcome both barriers. They have an extremely high theoretical specific energy (∼3500 Wh kg^−1^), making Li─O_2_ batteries attractive for use in weight‐sensitive applications [6, 7]. They are also free of any critical raw materials (e.g., cobalt), and thus could be produced at low cost and environmental impact, enabling widespread large‐scale usage [8]. However, several challenges currently prevent their commercial adoption, including short cycling lifetime and poor rate performance [9, 10].

Li─O_2_ batteries consist of a Li metal anode and an O_2_ cathode. On discharge, O_2_ is reduced via the oxygen reduction reaction (ORR) and reacts with Li^+^ ions in the electrolyte to form lithium‐oxygen compounds as the discharge product, typically Li_2_O_2_ [11, 12]. The discharge products are insulating and insoluble in typical non‐aqueous electrolyte solvents [12, 13], and as they form during discharge, they deposit on the electrode surface, thus preventing electron transfer from that area on the current collector to dissolved O_2_. This results in the passivation of the current collector as the cell discharges. To mitigate surface passivation effects and to increase available sites for the ORR to occur, a high specific surface area material is desired for this electrode. The porous, high surface area current collector is known as the air electrode. The air electrode must also have large enough, connected pores to prevent pore blockage by discharge product formation, allowing O_2_ diffusion from the external atmosphere through the porous structure, enabling discharge across the whole electrode, and thus maximising its utilisation. Porous carbon materials are typically used, as these satisfy the surface area, porosity, and conductivity requirements of the air electrode, while also benefiting from the natural abundance of carbon [14, 15].

**FIGURE 1:**
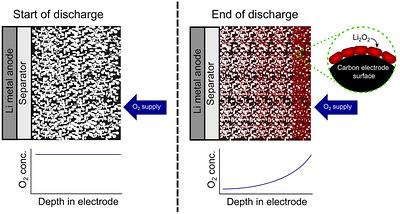
Schematic of Li─O_2_ battery illustrating surface passivation and O_2_ mass transport limitations. Left shows a pristine porous carbon air electrode (black) prior to discharge with a uniform O_2_ concentration profile, while right shows an air electrode with discharge products (red) accumulated at the O_2_‐side due to an O_2_ concentration gradient in the electrode, with an inset schematic demonstrating how Li_2_O_2_ passivates the carbon surface.

Both surface passivation and O_2_ mass transport are limiting factors on the discharge capacity of Li─O_2_ batteries, especially at high discharge rates. This is illustrated in Figure 1. Regarding surface passivation, larger particles of discharge product are generally preferred for high‐capacity batteries, as this increases the volume of discharge product formed before the surface is passivated, thus increasing capacity. As interfacial current density increases, discharge product particle size decreases/thin films form [16, 17, 18], and so discharge product particle size reduction is a potential source of capacity decrease at high discharge rates in Li─O_2_ batteries [19, 20]. Other factors, such as the donor number of the electrolyte solvent or the use of redox mediators, can also augment Li_2_O_2_ particle size [13, 21]. O_2_ mass transport is another potentially limiting factor. During discharge, O_2_ is consumed in the electrolyte. It is replenished by the dissolution of O_2_ from the external atmosphere and diffusion through the air electrode. At high rates of discharge, the replenishment of oxygen supply via diffusion of O_2_ through the electrolyte in the tortuous air electrode can become slower than the rate of consumption, developing an uneven O_2_ concentration profile in the electrode, with more O_2_ in solution closer to the O_2_ atmosphere (the “O_2_‐side” of the electrode) [10, 22, 23]. This non‐uniform O_2_ distribution promotes preferential formation of discharge products near the O_2_‐side, ultimately causing localized pore blockage and further hindering mass transport [24, 25, 26, 27].

Different failure modes of Li─O_2_ batteries have been tested through continuum modelling [28, 29, 30], but direct comparison of simulated and experimental discharge product distributions has been lacking. Some depth profiling of discharge product formation has been done in discharged air electrodes, but this has been limited in scope [26, 31, 32, 33, 34] or spatial resolution [10, 35, 36, 37, 38, 39]. In this work, limiting factors on Li─O_2_ discharge capacity were evaluated using a combination of scanning electron microscopy with energy dispersive X‐ray spectroscopy (SEM‐EDS) and X‐ray nano‐computed tomography (nano‐CT) to semi‐quantitatively map the distribution of discharge product through the air electrode. The experimental results have been compared with continuum modelling, validating the results derived from simulations. The results indicate that O_2_ mass transport is the primary limitation on capacity at high discharge rates in Li─O_2_ batteries, under the conditions probed here. These techniques can help determine the utilisation efficiency of the air electrode in metal‐air batteries.

## 2 Results and Discussion

### 2.1 Li─O_2_ Cell Discharge

**FIGURE 2:**
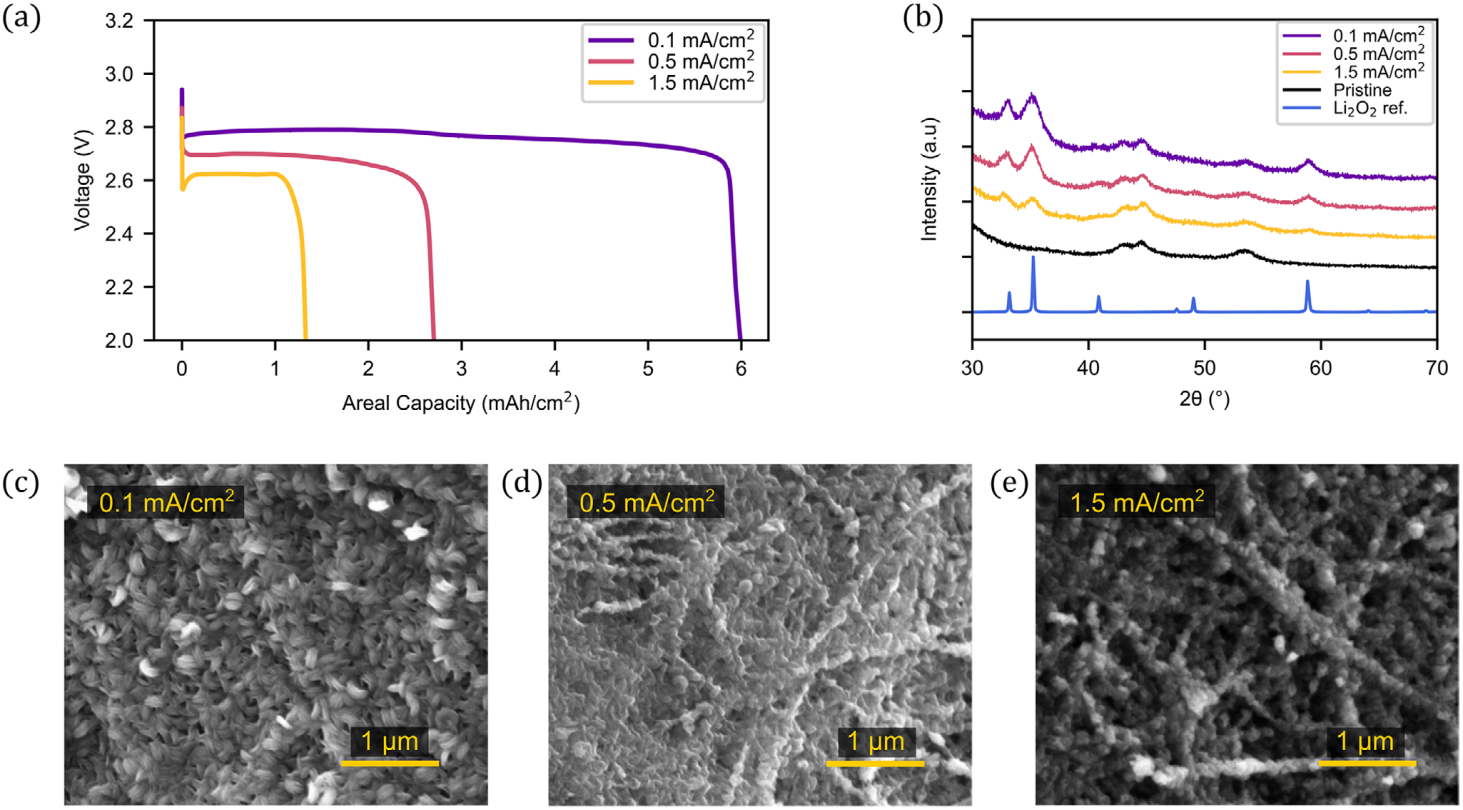
(a) Discharge curves of Li─O_2_ cells discharged at 0.1, 0.5, and 1.5 mA cm^−2^ (b) Powder X‐ray diffraction (pXRD) patterns of electrodes discharged at different rates, compared with pXRD pattern of the pristine electrode and a reference Li_2_O_2_ pXRD pattern (reference data from Zhuravlev et al. [40], CCDC 1690018) (c–e) SEM images of discharged Li─O_2_ battery air electrodes discharged at (c) 0.1 mA cm^−2^ (d) 0.5 mA cm^−2^ (e) 1.5 mA cm^−2.^

In order to evaluate the spatial distribution of discharge products within the air electrode, Swagelok‐type Li‐O_2_ cells were assembled with a Li‐metal anode, a binder‐free carbon nanotube‐mesh (CNT‐mesh) air electrode, and a 1 m LiTFSI in DMSO electrolyte. DMSO is a high donor number solvent that promotes the growth of larger Li_2_O_2_ crystals, thus reducing the effects of surface passivation, which is desirable for high‐capacity batteries [41, 42]. The CNT‐mesh electrode is compositionally uniform, and so the electrode structure will not affect the distribution of discharge products, i.e., there is no concern that uneven distribution of binder content in the air electrode would affect the distribution. Additionally, typical binders can be subject to degradation in Li─O_2_ batteries, limiting their utility [43, 44]. These Li─O_2_ cells were discharged at 0.1, 0.5, and 1.5 mA cm^−2^, with discharge curves shown in Figure 2a. With increasing discharge rate, two effects can be observed: overpotential increases and capacity decreases. Overpotential increases for Swagelok‐type cells have been ascribed mainly to an *iR* drop due to cell impedance and concentration polarisation due to slow O_2_ mass transport [45, 46]. There are two primary causes of the capacity reduction at high rates: smaller particles/thin films of discharge product forming and O_2_ starvation. Smaller Li_2_O_2_ particles reduce capacity as they reduce the volume of Li_2_O_2_ deposited on the CNT surface before passivation, thus lowering capacity [19, 20]. Slow O_2_ diffusion through the air electrode reduces capacity as it leads to Li_2_O_2_ formation primarily at the O_2_‐side, under‐utilising the rest of the electrode [10, 24, 25]. Powder X‐ray diffraction (pXRD) patterns of discharged air electrodes can be seen in Figure 2b, indicating a primary discharge product of Li_2_O_2_. The intensity of the Li_2_O_2_ peaks can be seen to decrease as discharge rates increase. This is primarily due to the reduction in discharge capacity seen as rates increase, resulting in less Li_2_O_2_ in the electrode to contribute to the observed peak intensities. The smaller crystallites formed at high rates may also reduce the diffraction peak intensities. Figure 2c–e shows SEM images of the electrode surfaces on the O_2_‐side after discharge, with particles of Li_2_O_2_ visible. As can be seen, particle size decreases with higher current density. At discharge current density of 0.1 mA cm^−2^, distinct Li_2_O_2_ particles are seen with a characteristic toroidal shape [22, 47], whereas higher discharge rates (i.e., 0.5 and 1.5 mA cm^−2^) generate smaller particles, forming a more conformal coating of the electrode surface. Higher magnification SEM images of the discharge products are shown in Figure S1. To elucidate if this reduced particle size is the primary cause of reduced capacity at increased discharge rates, or if it is due to O_2_ mass transport limitations, the distribution of discharge products through the air electrode is characterised using SEM‐EDS and X‐ray nano‐CT. If surface passivation was the primary limitation on discharge capacity, discharge products would be expected to be distributed relatively evenly throughout the electrode, whereas if O_2_ diffusion was the primary limitation, discharge products would be expected to be concentrated at the O_2_‐side.

### 2.2 Cross‐Sectional SEM‐EDS Characterization

We use cross‐sectional SEM‐EDS to investigate the distribution of discharge products vertically in the air electrode, and the resulting change in distribution for electrodes discharged at different rates. EDS characterises the elemental composition of the probed material by detecting characteristic X‐rays, which are emitted by elements in the sample that are excited by the electron beam as it is scanned across the sample during SEM. The relative amounts of characteristic X‐rays can be used to quantitatively determine the composition of the probed material at each point scanned during SEM imaging [48]. However, accurate quantification is non‐trivial, particularly for rough, layered samples such as Li_2_O_2_ on a carbon air electrode due to variations in X‐ray absorption [48]. Furthermore, quantification requires extensive standardisation or access to proprietary “standardless” databases, which are not always readily available [49]. As such, in this study, the oxygen Kα counts and carbon Kα counts were analysed to semi‐quantitatively determine the relative amount of discharge products to the carbon electrode. In the electrode, the carbon content is likely to be rather uniform across all points (as shown for the pristine material in Figure S2). Therefore, any increase in the oxygen‐to‐carbon ratio indicates an increased amount of oxygen‐containing discharge products, rather than a loss of carbon. The ratio of oxygen Kα X‐ray counts to carbon Kα X‐ray counts (O/C ratio) measured at each point on the electrode cross‐section during SEM‐EDS thus serves as a proxy measurement for the amount of discharge product across the electrode cross‐section. Factors such as surface roughness can impact this value, in particular, potentially decreasing the relative amount of the lighter element carbon Kα X‐ray counts due to higher absorption of lower energy photons in rough materials. However, previous work has shown this effect will be on the order of 10% from point‐to‐point [50, 51], particularly as oxygen and carbon are adjacent in Z number, so absorption differences are minor. Furthermore, the large number of points in each map will prevent any outlier points from being misinterpreted. SEM‐EDS analysis of battery cross‐sections has been previously employed to understand heterogeneities across the electrodes [52, 53, 54, 55].

To determine the relative amount of oxygen, and thus Li_2_O_2_, compared to carbon at each point in the electrode, the interaction volume of the electrons in the sample must be large enough to probe both the Li_2_O_2_ and the underlying carbon electrode. Monte‐Carlo simulations performed using CASINO [56] are shown in Figure S4, which indicates that for a film of Li_2_O_2_ of thickness 200 nm (the maximum size of observed Li_2_O_2_ particles seen in Figure 2c–e), there is significant excitation of the underlying carbon substrate. For thicker coverage of Li_2_O_2_, more carbon X‐rays will be absorbed, and so the O/C ratio will give a higher value than the amount of Li_2_O_2_ would otherwise; however, as this technique is intended to be semi‐quantitative and to capture trends in the distribution rather than absolute values, this variation should not affect any conclusions drawn from the interpretation of data. There is a limit for the quantification in this analysis for electrodes with an abundance of large Li_2_O_2_ crystals (>300 nm diameter) as very little carbon would be detected and the Li_2_O_2_ content would appear very large; however for the conditions under which mass transport limitations are most relevant (i.e. high rates of discharge), small Li_2_O_2_ particles tend to form due to the higher interfacial current densities [16, 17, 18]. Higher acceleration voltages could be used to increase the interaction volume of the electron beam to overcome this limitation, but this would, in turn, lead to other difficulties, such as increased beam damage [16] and less efficient oxygen and carbon inner‐shell ionisation [57].

**FIGURE 3:**
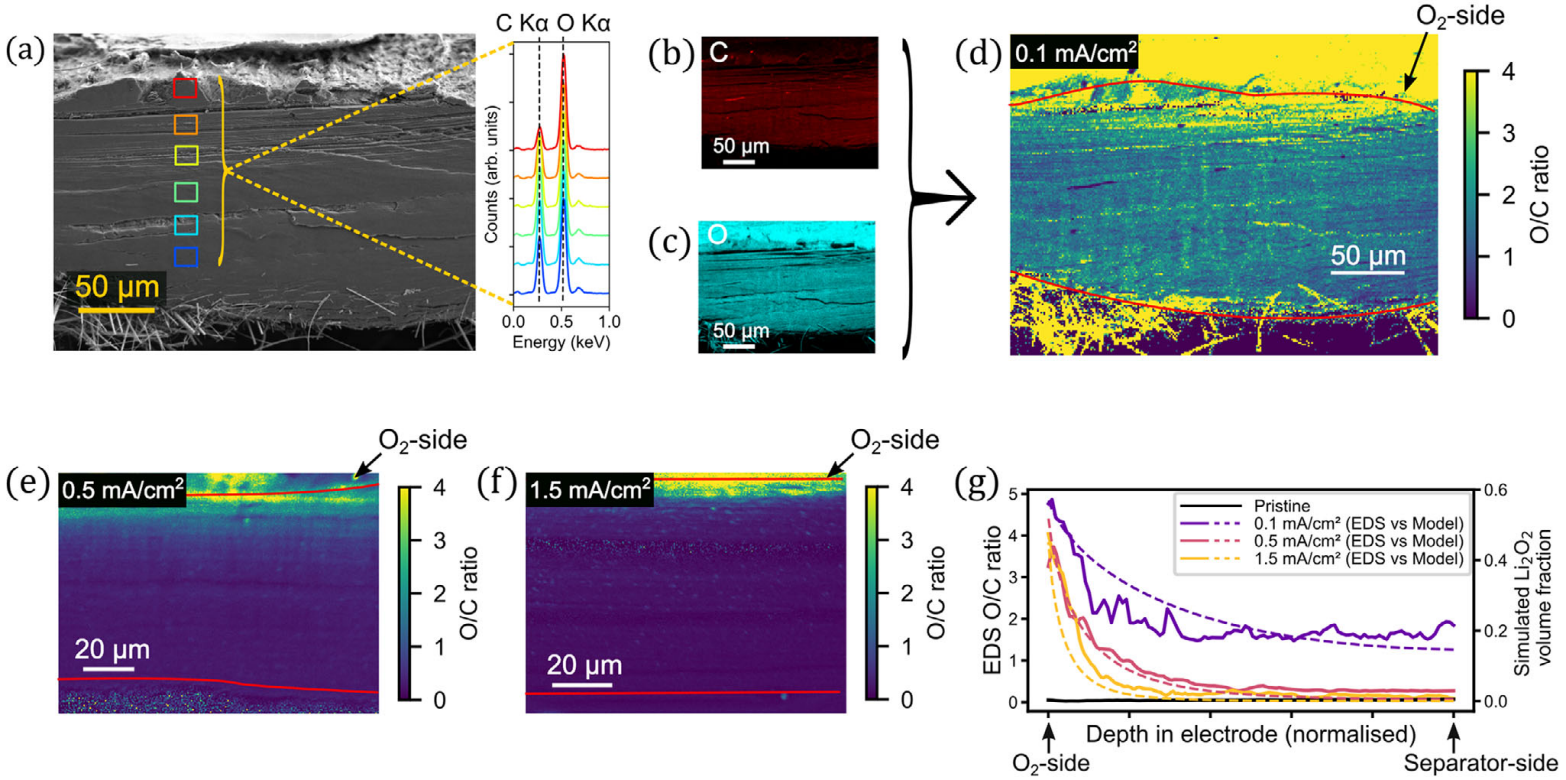
(a) SEM image of the cross‐section of an air electrode discharged at 0.1 mA cm^−2^ with EDS spectra from regions within the electrode, inset (b) EDS maps of carbon counts, and (c) oxygen counts. (d‐f) Map of O/C ratio of cross‐sections of electrodes discharged at (d) 0.1, (e) 0.5, and (f) 1.5 mA cm^−2^ (red lines indicate electrode upper and lower electrode edge) (g) Line profiles of O/C ratio data through depth of air electrodes discharged at different rates compared with simulated Li_2_O_2_ volume fraction from 1D continuum modelling.

A SEM image of the cross‐section of an air electrode discharged at 0.1 mA cm^−2^ is shown in Figure 3a, with example EDS spectra from local regions in the electrode inset. A small amount of electrode delamination can be seen due to the layered CNT structure of the electrode material delaminating during sample preparation (further details in the Experimental section). Maps of oxygen Kα and carbon Kα counts at this cross‐section are shown in Figure 3b,c, and the corresponding O/C ratio map can be seen in Figure 3d. O/C ratio maps of electrodes discharged at 0.5 and 1.5 mA cm^−2^ can be seen in Figure 3e,f, respectively, with the corresponding SEM images seen in Figure S3a,b. A cross‐sectional SEM image and EDS O/C ratio map of a pristine electrode is shown in Figure S2 for comparison, with very little oxygen detected, and that which is present is distributed uniformly across the electrode. This shows that the oxygen detected in the discharged electrodes is almost entirely due to discharge product formation. The small amount seen in the pristine electrode (corresponding to an O/C ratio of ∼0.08 compared to ∼4 for discharged electrodes) is likely due to some functional groups on the carbon nanotubes or some other strongly adsorbed oxygen‐containing species [58].

Figure 3d–f shows that for all test conditions in this study, the maximum point of discharge product formation is at the O_2_‐side. As discussed in Section 2.1, this indicates that O_2_ mass transport is the primary limitation on discharge capacity for all cells in this study. These discharge conditions have been replicated in a 1D continuum model of a Li─O_2_ cell, based on the work of Sahapatsombut et al. [28]. Details on continuum modelling are provided in the Supporting Information. Figure 3g shows average line profiles extracted from the O/C ratio maps in Figure 3d–f (Figure S2 for the pristine electrode) and compares them with simulated distributions of Li_2_O_2_. For all discharge rates, it can be seen in the electrodes that the O/C ratio initially decreases moving away from the O_2_‐side, before plateauing. The electrode discharged at 1.5 mA cm^−2^ exhibits a sudden decrease in O/C ratio before hitting a minimum value, whereas the electrodes discharged at 0.1 and 0.5 mA cm^−2^ have a more gradual decrease. The amount of discharge product formed deeper in the electrode, where the O/C ratio is relatively constant, is also dependent on discharge rate, with the electrode discharged at 0.1 mA cm^−2^ having a significantly higher O/C ratio than the electrode discharged at 0.5 mA cm^−2^, which in turn has a higher O/C ratio than the electrode discharged at 1.5 mA cm^−2^. This trend arises because, at higher current densities, oxygen is consumed more rapidly near the O_2_ interface, limiting its diffusion into the electrode interior and reducing Li_2_O_2_ formation deeper in the electrode. The observed distributions show that discharge products form primarily at the O_2_‐side under these discharge conditions, and thus give direct evidence supporting previous claims that the primary cause of cell death is poor mass transport of O_2_ due to slow diffusion and pore blockage at the O_2_‐side [10, 24, 25, 26]. This is consistent with simulated results, which demonstrate oxygen starvation leads to cell death, as can be seen in Figures S5–S7. These simulations show O_2_ concentration dropping to 0 at the end of discharge due to inadequate rates of diffusion, while abundant active surface area remains. For comparison, we have simulated a modified model to have rapid O_2_ diffusion and the formation of smaller Li_2_O_2_ particles, such that we simulate a primarily surface‐passivation‐limited regime. The results of these simulations can be seen in Figure S8. In this case, the monotonic decrease observed experimentally in Li_2_O_2_ content moving from O_2_‐side to the separator side is not seen; instead, the distribution is largely uniform, with a slight decrease in Li_2_O_2_ by the separator side. As this uniform Li_2_O_2_ profile is not seen experimentally, this corroborates the finding that O_2_ starvation, rather than surface area passivation, is the primary discharge termination mechanism under the conditions tested.

### 2.3 Correlative X‐Ray Nano‐CT Characterisation

X‐ray computed tomography (XCT) was employed in correlation with SEM‐EDS to investigate the spatial distribution of discharge products within the air electrode. XCT is a non‐invasive technique that allows for the imaging of the material's internal structure [59], and has been shown to be a powerful tool for understanding battery materials [60, 61, 62, 63, 64]. 2D radiographic projections are acquired over a 0–360° rotation and subsequently reconstructed by a mathematical algorithm to generate the cross‐sectional slices; the slice stack allows for the 3D visualization of the object. The reconstructed quantity for each voxel (each pixel in three dimensions) is the local value of the attenuation coefficient of the X‐rays. In the images shown later, this quantity is displayed in a grey scale, where the intensity of each voxel corresponds to the degree of X‐ray attenuation, which is primarily determined by the local material density and composition. Regions with higher X‐ray attenuation (denser phases) then appear brighter, while regions with lower X‐ray attenuation (less dense phases or voids) appear darker, approaching black (i.e., air) [65]. Specifically, here we used X‐ray nano‐CT, reaching a resolution of 300 nm voxel size using a nano‐CT system equipped with a LaB_6_ tip to reduce the physical size of the X‐ray source.

In discharged Li─O_2_ battery air electrodes, the accumulation of discharge products in the air electrode will cause a change in X‐ray attenuation, which can be used to map its presence. The electrode consists of a porous CNT network, the pores of which fill with discharge product as discharge progresses. As the voxel size is larger than the features in the electrode (individual Li_2_O_2_ particles, CNTs, pores with length scales ∼100 nm), the attenuation in each voxel will represent some average of the attenuation of the 3 different components (the attenuation coefficient of pores is zero, it is intermediate for carbon, and higher for Li_2_O_2_). This is known in XCT as the partial volume effect [59, 66]. The primary variation in attenuation in the present case will be caused by pores being filled with discharge products, as this is the replacement of a very low attenuation phase (pore) with a comparatively high attenuation phase (discharge product). As such, analysis of voxel grey‐values can give information on the relative amount of discharge product in each voxel in the characterised electrode. Other factors, primarily density heterogeneity in the pristine air electrode material, could also result in X‐ray attenuation variation. By correlating X‐ray nano‐CT data with SEM‐EDS measurements, attenuation variations can be confidently attributed primarily to changes in Li_2_O_2_ content, as discussed below. In this work, we implemented grey‐value analysis of X‐ray nano‐CT data to gain insight into the distribution of Li_2_O_2_ in the discharged air electrode.

**FIGURE 4:**
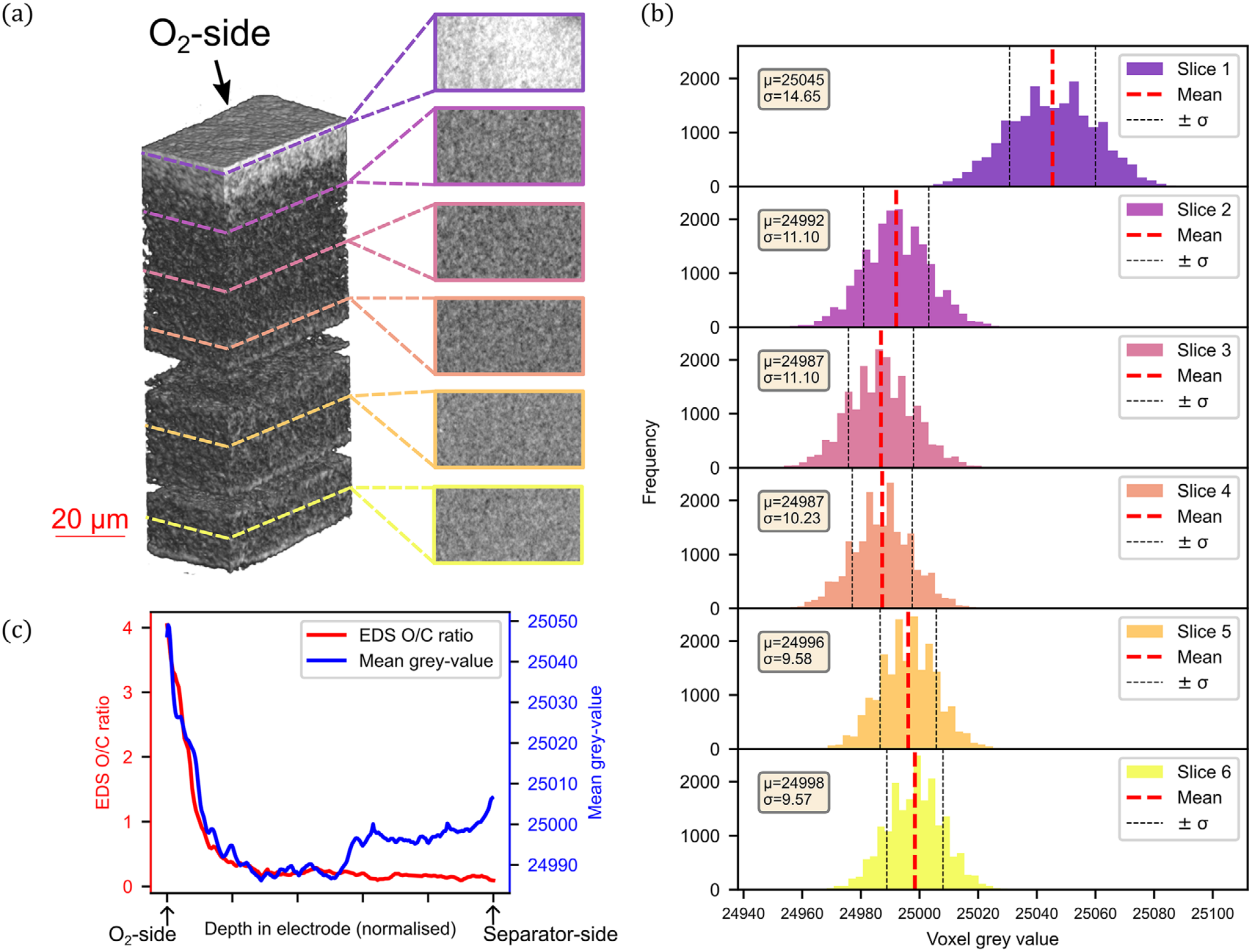
(a) X‐ray nano‐CT tomogram region of air electrode discharged at 1.5 mA cm^−2^ with representative slices shown (b) histograms of voxel grey‐values for representative slices (c) EDS O/C ratio and mean voxel grey‐value for all slices in 1.5 mA cm^−2^ electrode vs depth in electrode.

Figure 4 shows X‐ray nano‐CT analysis of a Li─O_2_ battery air electrode discharged at 1.5 mA cm^−2^ (as in Figure 3f). A region of interest (ROI) with dimensions 135 × 60 × 30 µm^3^ was extracted from the full tomogram (seen in Figure S9) for further statistical analysis. This ROI is shown in Figure 4a, with representative cross‐sectional slices spanning the thickness of the air electrode. The void regions between layers of the electrode are due to partial electrode delamination during the cutting of the sample for tomography, i.e., after the discharge products were formed, as was seen in Figure 3. The histograms of voxel grey‐values corresponding to these representative slices are shown in Figure 4b. As seen in Figure 3g, this electrode has a large amount of discharge products at the O_2_‐side, but their concentration drops off sharply deeper in the electrode. This results in increased mean X‐ray attenuation in slices at the O_2_‐side compared to deeper in the electrode. The mean voxel grey‐value is plotted for every slice (excluding those in void regions due to delamination) along with the O/C ratio for this electrode, shown in Figure 4c. There is a strong correlation with the mean voxel grey‐value and the O/C ratio, with both dropping to a minimum and remaining approximately constant at a depth of ∼20% into the air electrode (corresponding to a depth of ∼10 µm). This validates that electrode slicing does not significantly alter the spatial distribution of discharge products observed using SEM‐EDS. The slight increase in attenuation seen in this electrode in the CT analysis (mean voxel grey‐value) toward the separator side may be due to asymmetric swelling of the electrode when submerged in the electrolyte of the cell, resulting in density differences, or from inherent inhomogeneities in the pristine CNT sheet density. This is confirmed by X‐ray nano‐CT, which was performed on a pristine electrode, where comparable variation in attenuation was seen, as shown in Figure S10. Note finally that the grey level within each slice in Figure 4a is rather homogeneous, indicating the spatial distribution of the discharge product is homogeneous, likely due to the small size of the Li_2_O_2_ particles.

**FIGURE 5:**
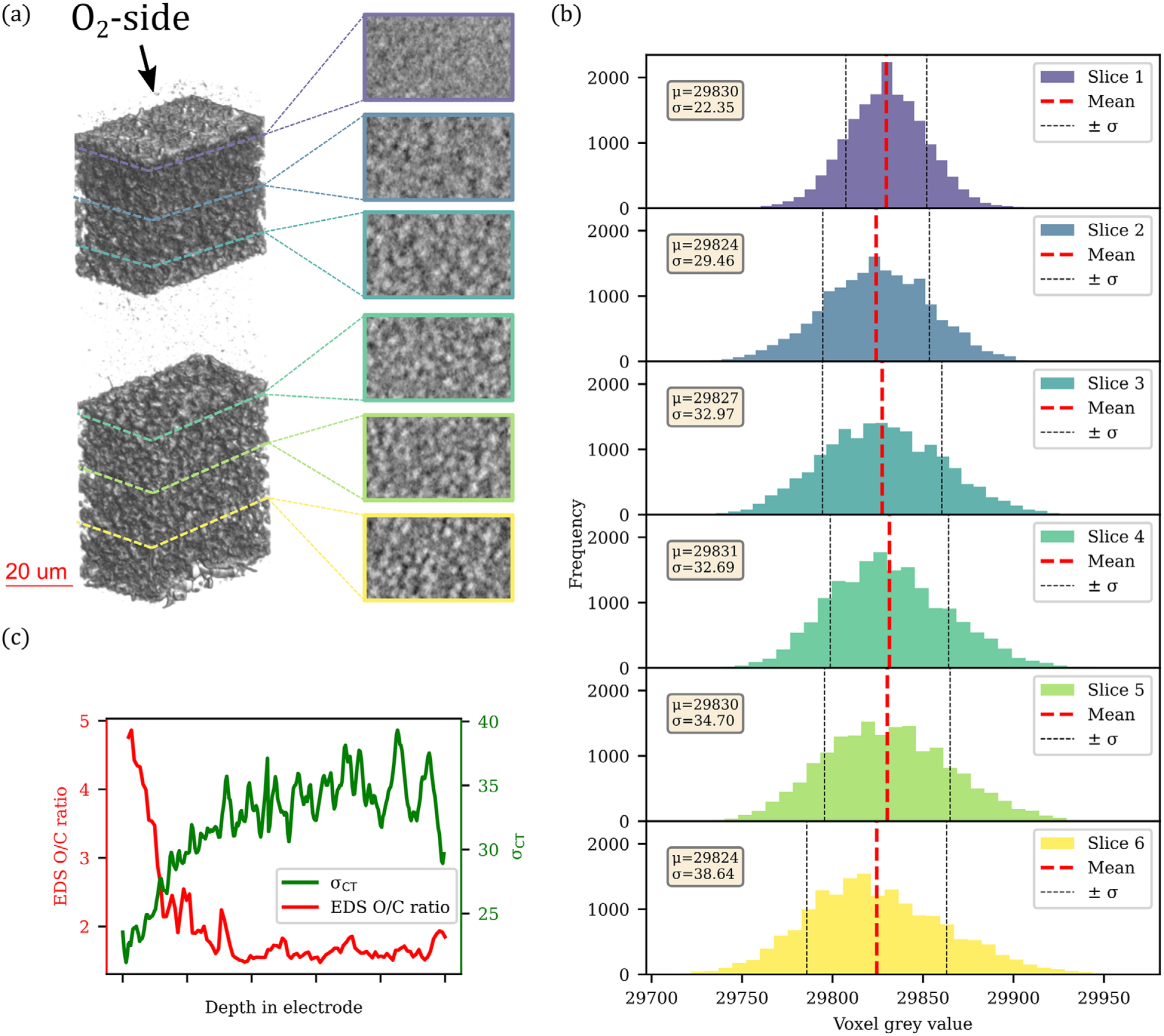
(a) X‐ray nano‐CT tomogram of a selected region within the air electrode discharged at 0.1 mA cm^−2^ with representative slices shown (b) histograms of voxel grey‐values for representative slices (c) EDS O/C ratio and standard deviation of voxel grey‐value for all slices in 0.1 mA cm^−2^ electrode vs depth in electrode.

Figure 5 shows X‐ray nano‐CT analysis of a Li─O_2_ battery air electrode discharged at 0.1 mA cm^−2^ (as in Figure 3d). A ROI with dimensions 150 × 50 × 30 µm^3^, extracted from the full tomogram (Figure S9), is shown in Figure 5a, with representative cross‐sectional slices shown through the electrode thickness. The corresponding histograms of voxel grey‐values in these slices are shown in Figure 5b. There is little variation in the mean voxel grey‐value in these slices going from the O_2_‐side to the separator side, in contrast to the large variation seen in the electrode discharged at 1.5 mA cm^−2^ (Figure 4). The low variation of the mean attenuation between slices (shown in Figure S11) is likely due to the relatively low signal‐to‐noise ratio in the reconstructed X‐ray attenuation, and so there is insufficient sensitivity to detect changes due to the smaller change in discharge product content through the thickness of the electrode. Specifically, for the electrode discharged at 0.1 mA cm^−2^, the O/C ratio measured by EDS changes by a factor of ∼2 through the electrode, whereas for the electrode discharged at 1.5 mA cm^−2,^ the O/C ratio changes by a factor of ∼10, as shown in Figure 3g. While the mean attenuation remains relatively constant through the electrode thickness, the standard deviation of the grey‐value grows progressively. This is because at the O_2_‐side there is a large amount of discharge products formed, and pores are effectively entirely filled, resulting in a nearly homogeneous mixture of discharge product and carbon, with minimal empty pore space. This compositional uniformity yields low grey‐value variability within the slices, reflected in a small standard deviation. Deeper within the electrode, where less discharge product is present, greater heterogeneity arises from the coexistence of regions with differing discharge product contents. This increased contrast among voxels produces a higher standard deviation in the corresponding slices.

The slices shown in Figure 5a exhibit distinct clusters, suggesting the formation of fewer, larger particles of discharge product. Simulations show that deeper in the electrode, there is lower interfacial current density due to lower O_2_ concentration, shown in Figures S5–S7. This in turn would lead to less particle nucleation and favour the growth of larger particles, as observed [67, 68].

The standard deviation of the pixel intensities in each slice in the 0.1 mA cm^−2^ electrode is plotted with the O/C ratio through the depth of the electrode in Figure 5c. Any slices that correspond to regions where the electrode has delaminated are excluded. The standard deviation of pixel intensities can be seen to be negatively correlated with the O/C ratio. This is consistent with the hypothesis that less discharge product formation (as indicated by the EDS data) gives a more porous electrode, and thus higher variation in pixel values. The shape of the curves is broadly similar, with an initial decrease/increase in the SEM‐EDS O/C ratio/X‐ray nano‐CT voxel grey‐value standard deviation before levelling off to be approximately constant, indicating that both techniques are semi‐quantitatively characterising the same variation in the spatial distribution of discharge products. As X‐ray nano‐CT is a non‐invasive technique that probes the interior of the electrode, this also validates that the slicing of the electrode does not significantly disrupt the distribution of discharge products along the cross‐section, which is characterised by SEM‐EDS.

## 3 Conclusions

In this work, we have demonstrated the usage of SEM‐EDS and X‐ray nano‐CT to characterise the distribution of discharge products in Li─O_2_ battery air electrodes, which is a key parameter determining the effective utilisation of the air electrode. We have validated the long‐standing hypothesis that the cause of sudden death in Li─O_2_ batteries is oxygen starvation, leading to the production of discharge products predominantly at the O_2_‐side and under‐utilisation of the rest of the electrode, at least under the conditions tested in this work. These results were compared with simulations based on previous work in the literature to model these cells. Due to the nanoscale length of features in the electrode, in the X‐ray nano‐CT data, the partial volume effect was leveraged to determine the variation of discharge product content throughout the electrode, as direct phase segmentation of the three components present in the electrode (CNT, discharge product, and pores) would require extremely high spatial resolution and signal‐to‐noise ratio. Statistical analysis of voxel grey‐value distribution across tomographic slices revealed discharge product distributions consistent with SEM‐EDS elemental mappings, thereby validating the imaging and quantification approach.

SEM‐EDS and XCT are relatively facile to implement and can be performed entirely on widely available, laboratory‐based equipment. Understanding the discharge product distribution in Li─O_2_ batteries can be used to determine the battery's failure mechanism and thus aid in the development of novel electrolytes and electrode structures. For example, for a dimethoxyethane‐based electrolyte, there would be both faster O_2_ transport (due to a higher diffusion coefficient) and smaller Li_2_O_2_ particle formation (due to low donicity) [69, 70], increasing the rate of surface passivation. The impact of changing these parameters are simulated in Figure S8, and would be expected to result in a more uniform distribution of Li_2_O_2_ in the air electrode. However, these parameters are highly interdependent [18] and so experimental investigation across a wide range of electrolytes is required to verify these simulations. These techniques can additionally be applied after charging the cell to identify spatial heterogeneity in Li_2_O_2_ decomposition or decomposition product formation. The impact of various electrolyte parameters on the Li_2_O_2_ distribution on discharged and charged Li─O_2_ batteries is an area that we are investigating further.

## 4 Experimental Section

### 4.1 Li─O_2_ Battery Discharge and Air Electrode Recovery

Swagelok‐type Li─O_2_ cells were used in this study. Cell assembly was performed in an argon‐filled glovebox (H_2_O < 1 ppm, O2< 0.1 ppm). They were assembled by placing a Li disc on a steel spacer, which is then placed on a plunger on one side of the cell. A Whatman glass fibre (rinsed in ethanol and acetone, dried at 120°C under vacuum) was added, followed by the CNT sheet air electrode (Tortech, dried at 120°C under vacuum). The air electrode has a diameter 10 mm (area = 0.79 cm^2^) and a mass of 2.2 ± 0.1 mg (mass loading = 2.8 mg cm^−2^). The CNT sheet is made via floating‐catalyst chemical vapour deposition and subsequent winding of the CNTs onto a drum, resulting in the layered structure seen [71]. 150 µL of 1 M LiTFSI (Sigma Aldrich, 99.99% trace metals basis, dried at 120°C under vacuum) in DMSO (Sigma Aldrich, ≥99.9% anhydrous, dried using 4 Å molecular sieves (Sigma Aldrich)) electrolyte was added, followed by a steel mesh current collector, which allows for gas diffusion into the cell. A hollow top plunger with a Swagelok Quick Connect valve was then placed in, and pressure was applied while the cell was screwed shut to seal the cell prior to removal from the glovebox. A small oxygen tank of volume ∼ 100 cm^3^ with a Swagelok Quick Connect body attached was filled with 1.5 bar O_2_. The Li─O_2_ cell was attached to this tank and discharged galvanostatically at the desired current density until cell potential reached 2 V using an Arbin BT‐2000 potentiostat. After discharge, the Li─O_2_ cell was evacuated and brought into the glovebox. The cell was disassembled, and the air electrode was removed. The air electrode was rinsed twice with dimethoxyethane (DME) (degassed, dried using 3 Å molecular sieves (Sigma Aldrich)) to remove electrolyte residue. The air electrode is then dried under vacuum at room temperature and stored in the glovebox for further characterisation.

### 4.2 SEM‐EDS

To characterise the cross‐section of the cathode, a small portion is sliced off with a razor. The exposed side is then mounted on an appropriate SEM stub such that the cross‐section is pointing upward. This is illustrated in Figure S12. This is then transferred to the SEM chamber using an airtight Kammrath and Weiss transfer module to prevent exposure to air. The SEM used was a TESCAN CLARA2, and the EDS detector was an Oxford Instruments MaxN silicon drift detector, 80 mm^2^ active area. Beam voltage and current were 5 kV and 3 nA, respectively, for EDS measurements.

### 4.3 Powder X‐Ray Diffraction

Electrodes were sealed in Kapton film to prevent air exposure during XRD measurement. A Panalytical Empyrean diffractometer was used with a Cu Kα source (λ = 1.542 Å, 40 kV, 40 mA).

### 4.4 X‐Ray Nano‐CT

A slice of electrode was sealed in polyimide tubing (Cole‐Parmer, USA. Dimensions: ID 1 mm, OD 1.1 mm) using epoxy resin to prevent air exposure. This is illustrated in Figure S12. 3D tomographic characterization of the air electrodes was performed using an EasyTom Nano system (Rx Solution, Chavanod, France). The instrument is equipped with a high‐resolution CCD detector optimized for imaging low‐density materials at moderate X‐ray energies. A LaB_6_ (lanthanum hexaboride) filament source was employed to provide a high‐brightness X‐ray beam with a spot size of 300 nm, reducing the amount of geometric blur. Tomographic datasets were acquired at a voxel size of 300 nm, with an accelerating voltage of 80 kV, a beam current of 200 µA, and an exposure time of 1.6 s per projection. A total of 1504 radiographic projections were recorded over a full rotation. The reconstruction consists of 16‐bit images. Due to the relatively low density and low X‐ray attenuation of the electrode materials, no beam filters were applied during acquisition.

## Funding

This research was funded by the Engineering and Physical Sciences Research Councilvia the NanoDTC, grant no. EP/S022953/1, and by Cambridge Display Technologies Ltd. M.M. and E.M. acknowledge funding from the French Agency for National Research (Agence Nationale de la Recherche). Project name:  Dynamobat, reference: ANR‐22‐CE42‐0025. M.D.V. acknowledges funding from European Research Council (Consolidator Grant, MIGHTY, 866005). I. T. acknowledges support from a Beatriz Galindo Senior Fellowship (BG22/00148) funded by the Ministerio de Universidades (Spain), as well as from the project PID2024‐163065OB‐I00 funded by MICIU/AEI, and from the Xunta de Galicia (ED431F 2025/038).

## Conflicts of Interest

The authors declare no conflicts of interest.

## Supporting information




**Supporting File**: smtd70699‐sup‐0001‐SuppMat.docx.

## Data Availability

The data that support the findings of this study are openly available in Symplectic Elements at https://doi.org/10.17863/CAM.124547. The code for the PyBaMM‐based simulated Li‐O2 battery is available at https://github.com/elbee99/mapping_Li‐O2_battery_sim.
