# Supplementary material for: High‐Resolution Mapping of Discharge Product in Li─O2 Batteries
Source: Small Methods. 2026 May 7;10(14):e70699. doi: 10.1002/smtd.70699 (PMC13397231; doi:10.1002/smtd.70699)
Supplement: Supplementary file 1 — Supporting File: smtd70699‐sup‐0001‐SuppMat.docx. [file SMTD-10-e70699-s001.docx]

Supporting Information

High-resolution mapping of discharge product in Li-O_2_ batteries

Laurence F. Brazel, Margherita Martini, Eric Maire, Arnaud Demortière, Michael De Volder, Clare P. Grey, Israel Temprano*

**Supplementary SEM-EDS**

**
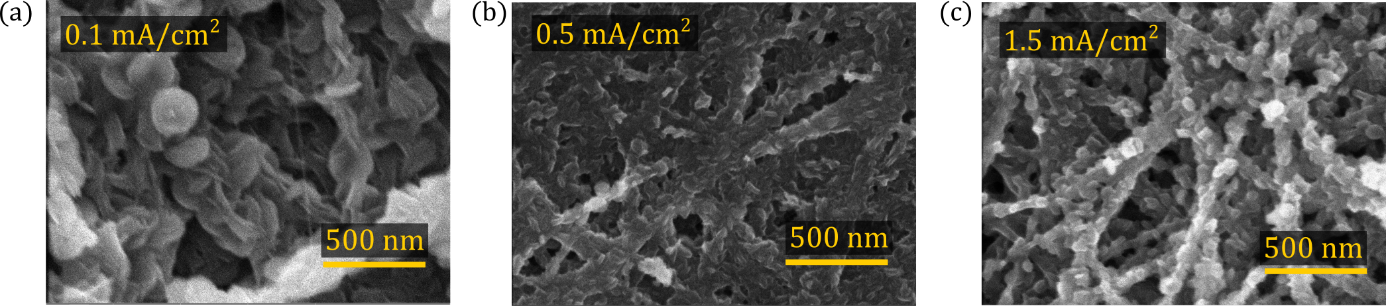
**

**Figure S1.** SEM images of discharge products on air electrode of Li-O_2_ cells discharged at (a) 0.1 mA cm^-2^ (b) 0.5 mA cm^-2^ and (c) 1.5 mA cm^-2^


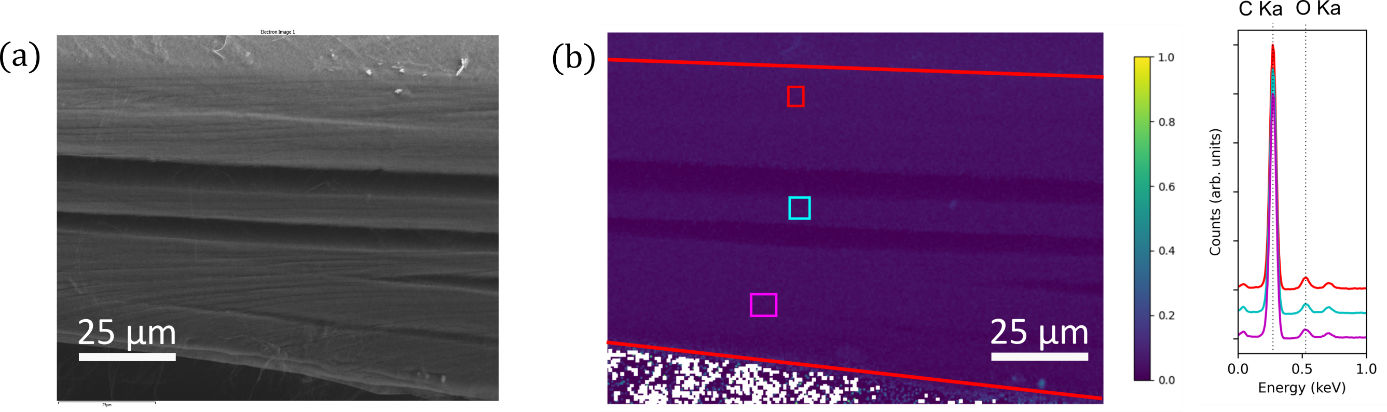


**Figure S2.** (a) SEM images of cross-section of pristine air electrode (b) EDS O/C ratio map with local spectra shown


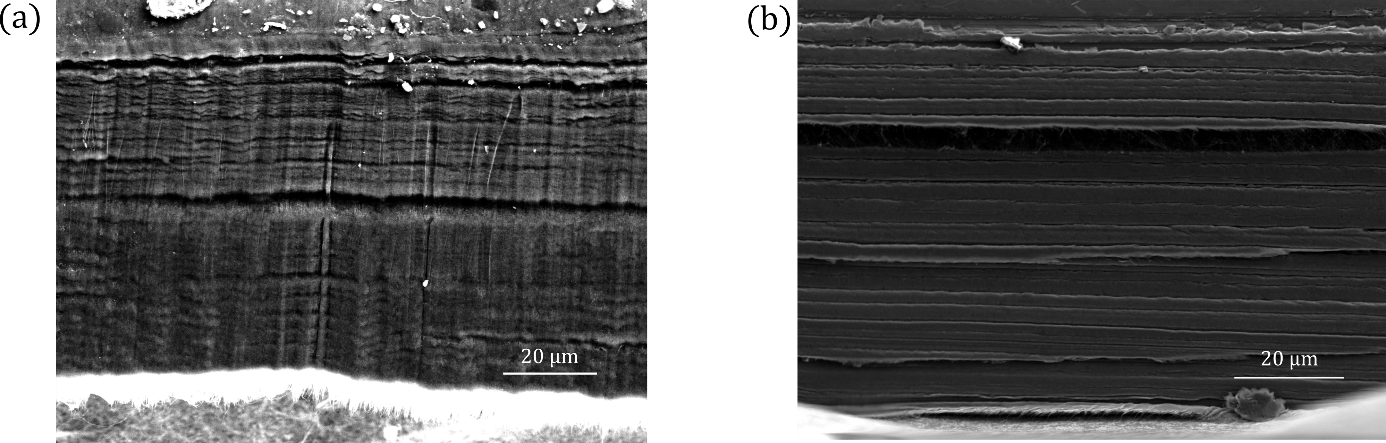


**Figure S3.** SEM images of cross-section of air electrodes discharged at (a) 0.5 mA cm^-2^ and (b) 1.5 mA cm^-2^

The pristine electrode was placed in a cell with electrolyte, rested without discharge, before removal, rinsing and drying in order to accurately compare pristine and discharged electrodes.

**Simulation of electron interaction volume**

***
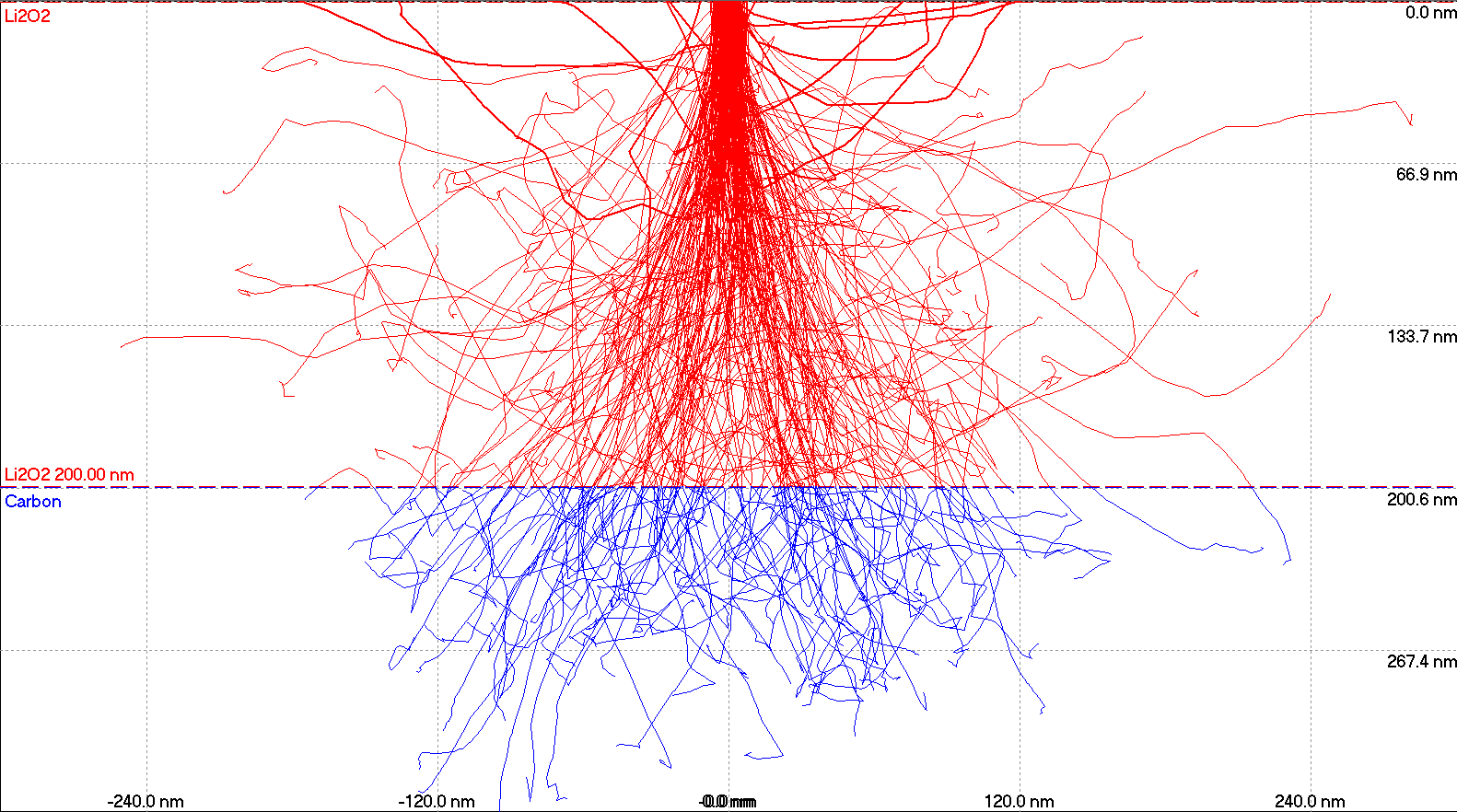
***

***
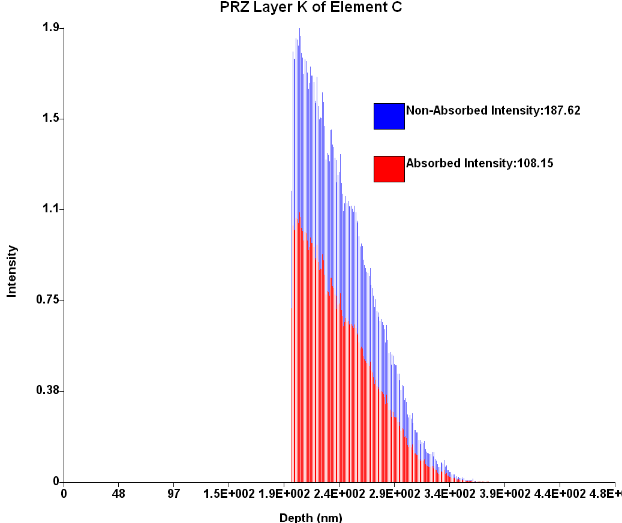

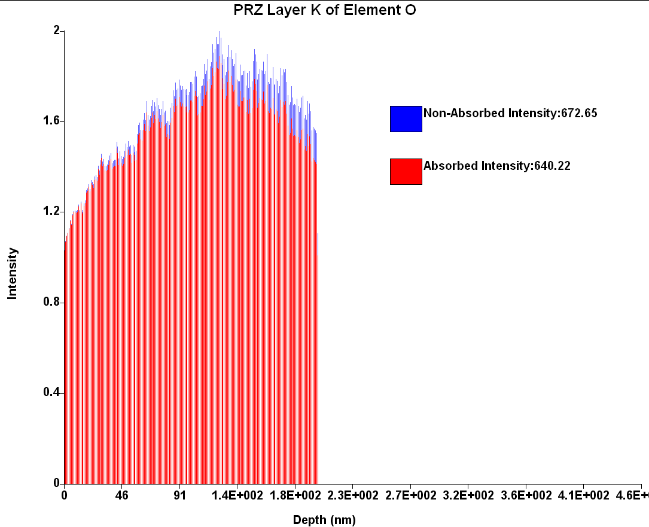
***

**Figure S4.** (a) electron trajectories at 5 kV acceleration in 200 nm of Li_2_O_2_ on carbon substrate simulated using CASINO (b,c) depth of emitted (blue) and detected (red) (b) oxygen Kα and (c) carbon Kα X-rays from sample

CASINO v.2.4.8.1 was used to simulate the interaction volume of electrons in a Li_2_O_2_ film on a carbon substrate. In our electrodes in most regions there will be significantly less Li_2_O_2_ and more pores, so the interaction volume will larger.

CASINO uses Monte-Carlo simulations to predict electron trajectories. The settings used in the software were:

- Sample:
  - Layer, composition Li2O2, density = 2.26 g cm^-3^, 200 nm thickness
  - Substrate, composition C, density = 2.62 g cm^-3^
- Physics models (all default):
  - Total Cross Section: Mott by Interpolation
  - Partial Cross Section: Mott by Interpolation
  - Effective Section Ionisation: Casnati
  - Ionisation Potential: Joy and Luo [1989]
  - Random Number Generator: Press et al. [1986]
  - Directing Cosin: Drouin [1996]
  - dE/dS Calculation: Joy and Luo [1989]

**Continuum modelling of Li-O_2_ battery**

This continuum model is based largely on the work of Sahapatsombut et al. [1] as mentioned in the main text. This model was implemented in PyBaMM [2]. The model is briefly described here.

Electrochemical reactions

The cell is modelled as a half-cell vs Li with negligible overpotentials arising from the Li anode. The cathodic reaction is:

$$2Li^{+}(sol)+O_{2}(sol)+2e^{-}\to Li_{2}O_{2}(s)$$

( 1 )

The rate of this reaction is calculated via Butler-Volmer kinetics as:

$$j=kc_{Li+}^{2}c_{O2}\left( \exp\left( \frac{F\eta}{2RT} \right)-\exp\left( -\frac{F\eta}{2RT} \right) \right)$$

( 2 )

where *j* is interfacial current density, *k* is the rate constant of the electrochemical reaction, $c_{Li+/O2}$ is the concentration of Li^+^ and O_2_ in the electrolyte, respectively, *F* is Faraday’s constant, *η* is the overpotential, *R* is the molar gas constant, *T* is temperature. The overpotential *η* is calculated as:

$$\eta=\phi-\phi_{e}-E^{0}$$

( 3 )

where *ϕ* is the positive electrode working potenital, *ϕ_e_* is the electrolyte potential and *E^0^* is the equilibrium potential of Li_2_O_2_ formation (2.96 V).

Current calculations

The current density in the positive electrode is calculated via:

$$i= -\sigma\nabla\phi$$

( 4 )

where *σ* is the electrode conductivity. The electrolyte current density, meanwhile, is calculated as:

$$i_{e}=\kappa\nabla\phi_{e}-\left( \frac{2RT\kappa}{F} \right)\left( 1-t^{+} \right)\left( 1+\frac{\partial ln f}{\partial\ln c_{Li+}} \right)\left( \nabla\ln c_{\mathrm{Li}+} \right)$$

( 5 )

where *κ* is the electrolyte conductivity, *t_+_* is the transference number of Li^+^, and *f* is the activity coefficient of LiTFSI salt.

Mass transport and conservation

The electrode is treated as a continuous medium with porosity *ε* and so the mass conservation equation for a species *i* in solution can be expressed as:

$$\frac{\partial\left( \varepsilon c_{i} \right)}{\partial t}-\nabla\cdot N_{i}+r_{i}$$

( 6 )

where *N_i_* is the molar flux of species *i* and *r_i_* is the rate of consumption/generation of species *i*. For Li^+^ the flux is defined as:

$$N_{Li+}=-D_{Li+,eff}\nabla c_{Li+}+\frac{i_{e}t_{+}}{F}$$

( 7 )

Where *D_Li+,eff_* is the effective diffusion coefficient for Li^+^ and for O_2_ the flux is defined as:

$$N_{O2}=-D_{O2,eff}\nabla c_{O2}$$

( 8 )

The rate of consumption/generation of a species is determined by Faraday’s law:

$$r_{i}=\frac{s_{i}aj}{2F}$$

( 9 )

where *s_i_* is the stoichiometric coefficient in equation (1) of the species being consumed/generated (+2, +1 and -1 for Li^+^, O_2_ and Li_2_O_2_, respectively), and *a* is the active surface area of the electrode. As *j* is negative, a positive value of *s_i_* indicates species consumption and a negative value of *s_i_* indicates species generation.

Porosity and surface area evolution

The volume fraction of Li_2_O_2_ is calculated from the generated concentration:

$$\varepsilon_{Li2O2}=\frac{c_{Li2O2}M_{Li2O2}}{\rho_{Li2O2}}$$

( 10 )

where *M_Li2O2_* is the molar mass of Li_2_O_2_ and *ρ_Li2O2_* is the density of Li_2_O_2_. The porosity then evolves during discharge via:

$$\varepsilon=\varepsilon_{0}-\varepsilon_{Li2O2}$$

( 11 )

where *ε_0_* is the initial porosity of the electrode. The diffusion coefficients evolve via the Bruggeman relation such that:

$$D_{eff,i}=D_{i}\varepsilon^{1.5}$$

where *D_i_* is the species initial diffusion coefficient.

The active surface area is passivated by the growth of Li_2_O_2_, and this is modelled via the equation:

$$a=a_{0}\left( 1-\left( \frac{\varepsilon_{Li2O2}}{\varepsilon_{0}} \right)^{0.5} \right)$$

( 12 )

where *a_0_* is the initial surface area per unit volume.

Initial and boundary conditions

Cell has a separator, which is inert, and electrode domain. The initial concentration of Li^+^ and O_2_ are defined as *c_Li+,0_* and *c_O2,0_* respectively. They are defined to be uniform at all points initially. During discharge, at the left (O_2_ source) side, the concentration of oxygen is held to *c_O2,0_* and at the right (Li^+^ source) side, the concentration of Li^+^ is held to *c_Li+,0_*. The left boundary of electrode current density is defined such that $i=\frac{I_{app}}{A\sigma}$ where *I_app_* is the applied cell current density and *A* is the cross-sectional area of the cell.

Parameter values

| **Parameter** | **Value** | **Unit** | **Symbol** |
| --- | --- | --- | --- |
| Initial surface area per unit volume | 2 × 10^7^ | m^-1^ | *a* |
| Separator thickness | 50 × 10^-6^ | m | *L_s_* |
| Electrode thickness | 200 × 10^-6^ | m | *L_e_* |
| Electrode cross-sectional area | 1 × 10^-4^ | m^2^ | *A* |
| Electrode conductivity | 1 | S m^-1^ | *σ* |
| Li^+^ diffusion coefficient | 2.11 × 10^-9^ | m^2^ s^-1^ | *D_Li+_* |
| O_2_ diffusion coefficient | 0.7 × 10^-9^ | m^2^ s^-1^ | *D_O2_* |
| Li^+^ transference number | 0.25 | - | *t_+_* |
| Activity dependence | -1 | - | $\frac{\partial\ln f}{\partial\ln c_{Li+}}$ |
| Electrolyte conductivity | 0.1 | S m^-1^ | *κ* |
| Faraday constant | 96485 | C mol^-1^ | *F* |
| Molar gas constant | 8.314 | J mol^-1^ K^-1^ | *R* |
| Temperature | 298.15 | K | *T* |
| Electrochemical rate constant | 6 × 10^-12^ | A m^7^ mol^-3^ | *k* |
| Open-circuit potential | 2.96 | V | *E^0^* |
| Initial Li^+^ concentration | 1000 | mol m^-3^ | *C_Li+,0_* |
| Initial O_2_ concentration | 3.7 | mol m^-3^ | *C_O2,0_* |
| Li_2_O_2_ molar mass | 4.588 × 10^-4^ | kg mol^-1^ | *M_Li2O2_* |
| Li_2_O_2_ density | 2310 | kg m^-3^ | *ρ_Li2O2_* |
| Initial porosity | 0.6 | - | *ε_0_* |

Table S1: parameters used in simulation of Li-O_2_ battery model

Detailed simulation results
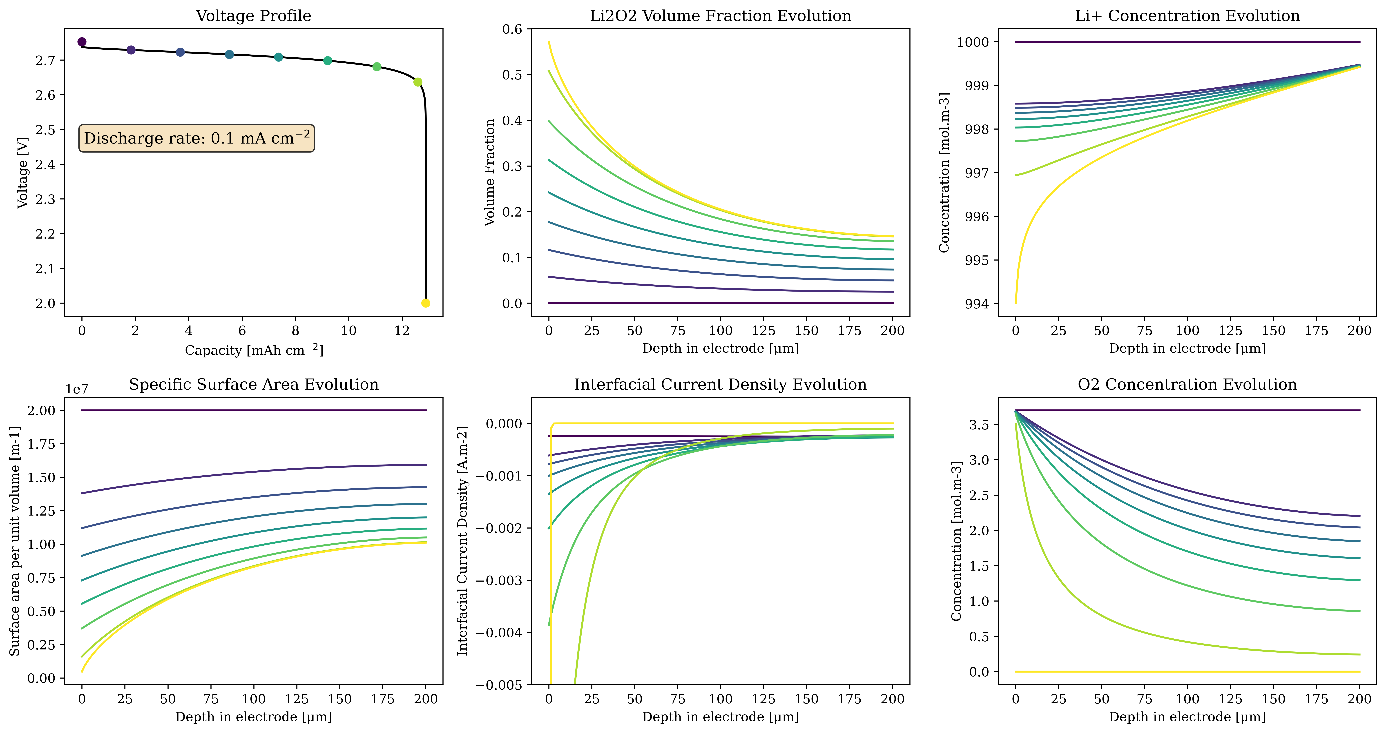


**Figure S5.** Results of spatial and temporal variation of selected simulated variables in Li-O_2_ battery discharged at 0.1 mA cm^-2^

**
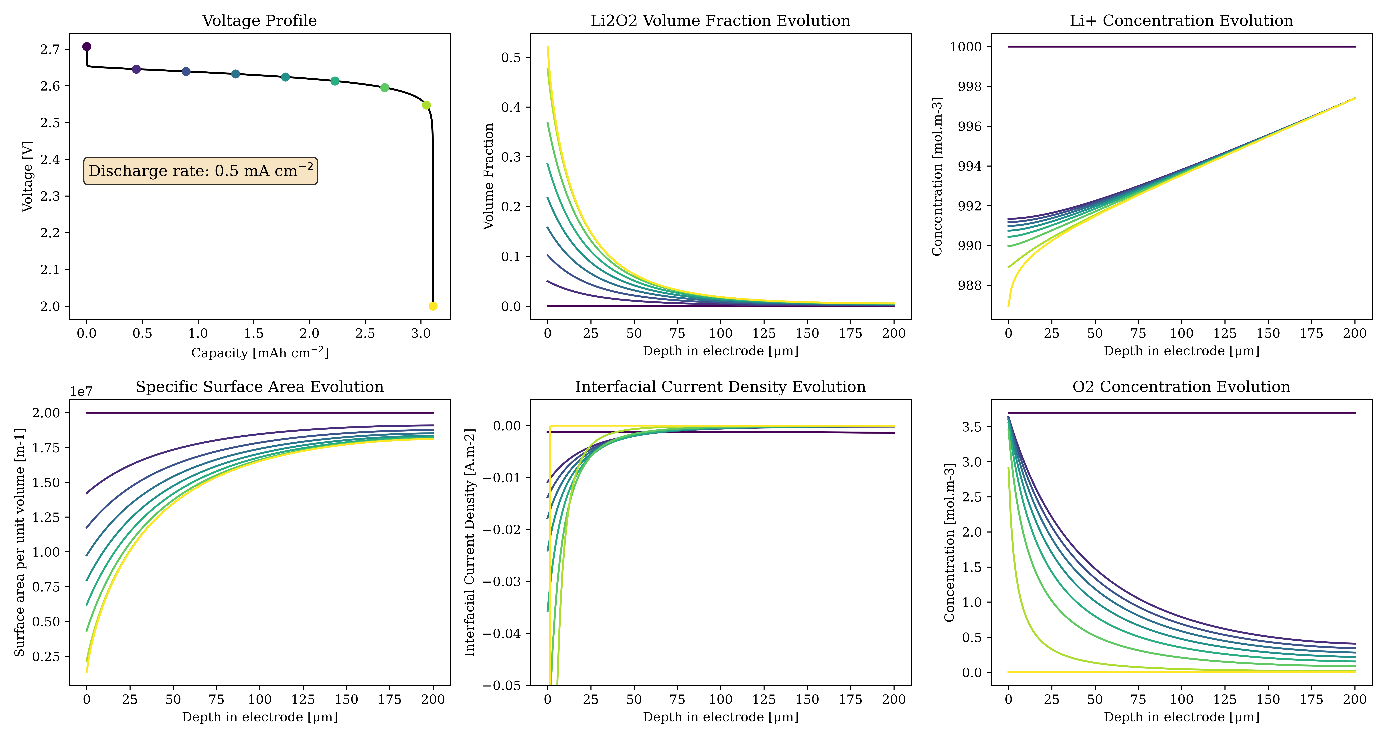
**

**Figure S6.** Results of spatial and temporal variation of selected simulated variables in Li-O_2_ battery discharged at 0.5 mA cm^-2^

**
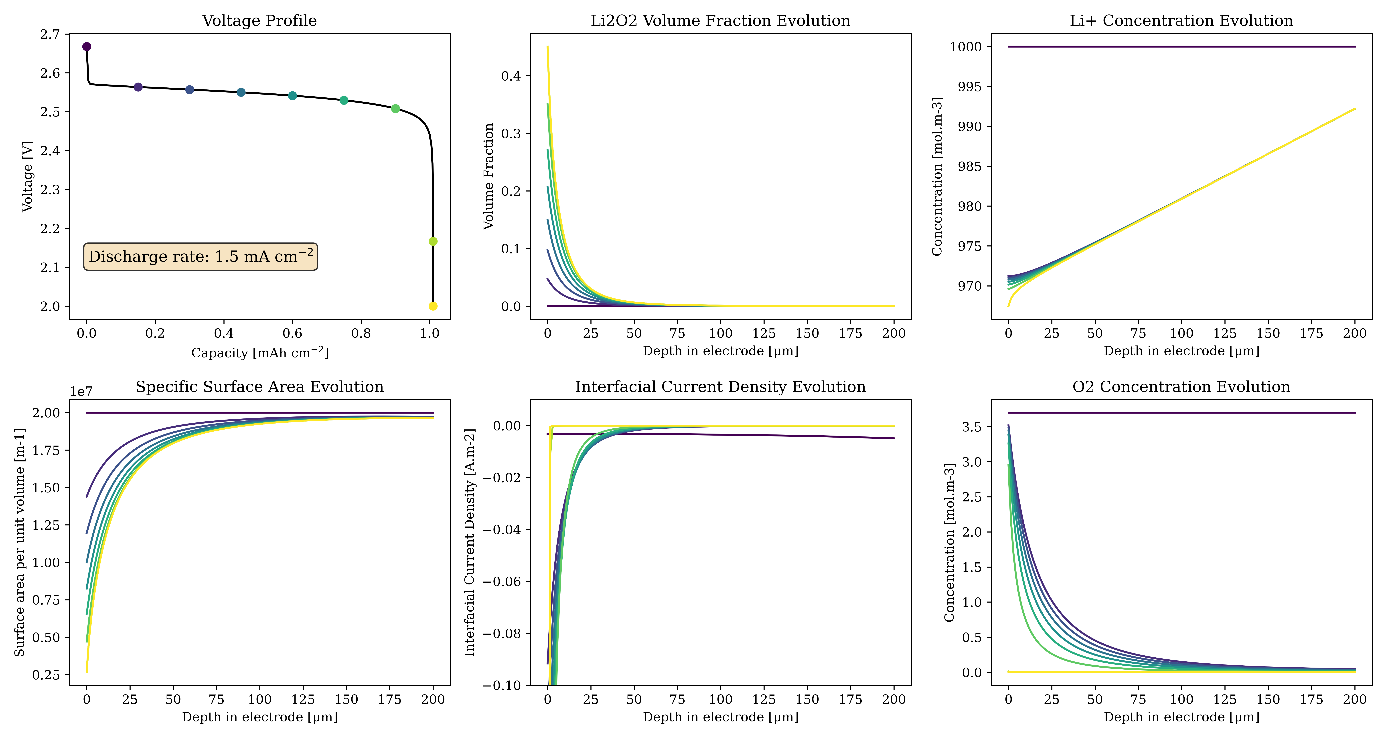
**

**Figure S7.** Results of spatial and temporal variation of selected simulated variables in Li-O_2_ battery discharged at 1.5 mA cm^-2^

The simulated results all are consistent with the hypothesis that O_2_ starvation is the primary cause of sudden death in Li-O_2_ batteries. This is seen as the O_2_ concentration drops to 0 at the end of discharge in the majority of the electrode, and so it is impossible to sustain discharge at the required rate. This is exacerbated by pore-blocking at the O_2_ side, as it can be seen in all electrodes that porosity reduces significantly as Li_2_O_2_ fills the pores predominantly at the O_2_-side. Surface passivation is not as significant a limitation as can be seen that a large amount of surface area remains active at the end of discharge.

Surface passivation-limited model

In order to simulate conditions under which surface passivation is limiting, we made two changes. We increased *D_O2_* to from 0.7 × 10^-9^ m^2^ s^-1^ to 5 × 10^-9^ m^2^ s^-1^, and altered equation (12) to:

$$a=a_{0}\left( 1-\left( \frac{\varepsilon_{Li2O2}}{\varepsilon_{Li2O2,max}} \right)^{0.5} \right)$$

( 13)

Where $\varepsilon_{Li2O2,max}$ indicates the maximum volume fraction of Li_2_O_2_ which can form. If $\varepsilon_{Li2O2,max}=\varepsilon_{0}$, we simulate complete pore filling with sufficient Li_2_O_2_ formation, whereas for small values of $\varepsilon_{Li2O2,max}$, it represents thin films of Li_2_O_2_ forming, which cannot fill the pores of the electrode structure. For this example, we selected $\varepsilon_{Li2O2,max}=0.2$. Below are **
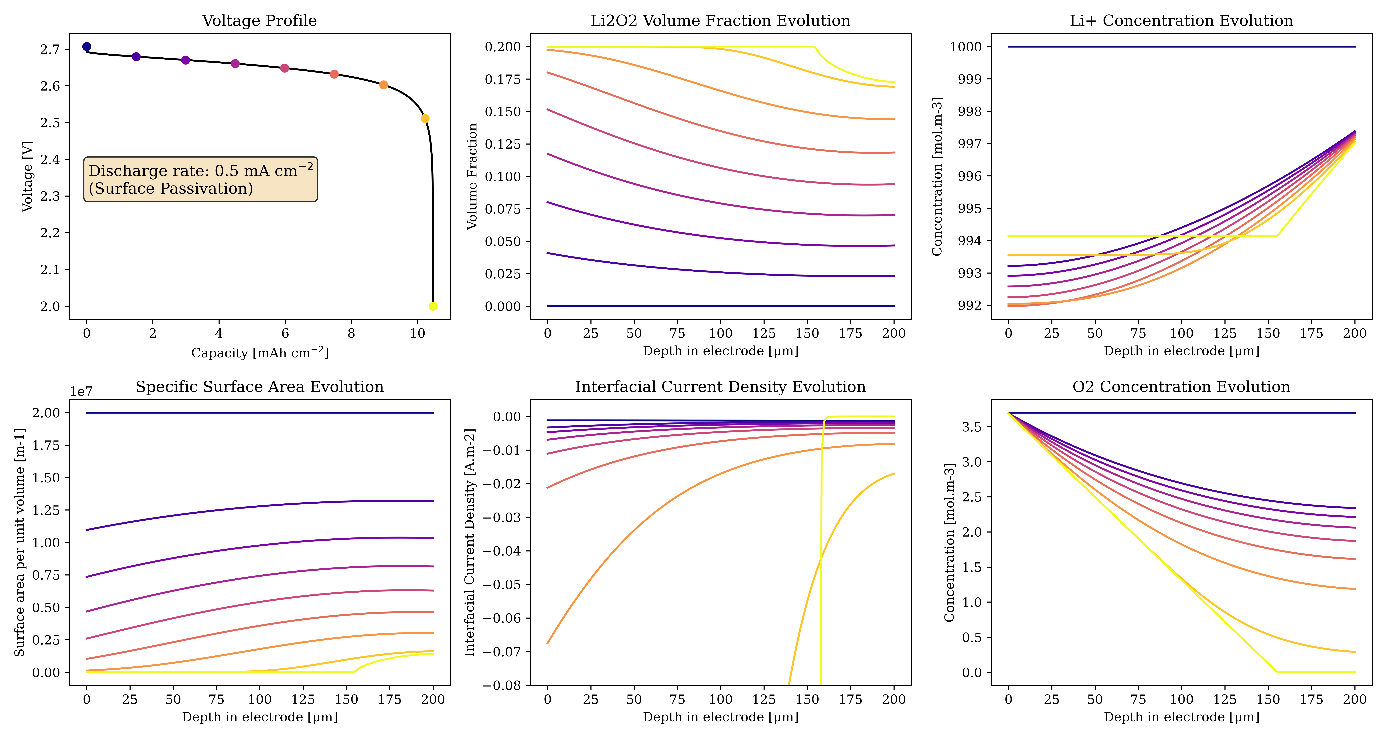
**simulated results for these conditions:

**Figure S8.** Results of spatial and temporal variation of selected simulated variables in passivation-limited Li-O_2_ battery discharged at 0.5 mA cm^-2^

In this regime, in which O_2_ transport is fast and Li_2_O_2_ deposition is limited, it can be seen that the Li_2_O_2_ distribution is largely uniform in the electrode, in contrast to what is seen experimentally. This coincides with active surface area being almost fully passivated This further corroborates that in the cells studied, O_2_ transport was thr primary limitation on discharge capacity, rather than surface area passivation.


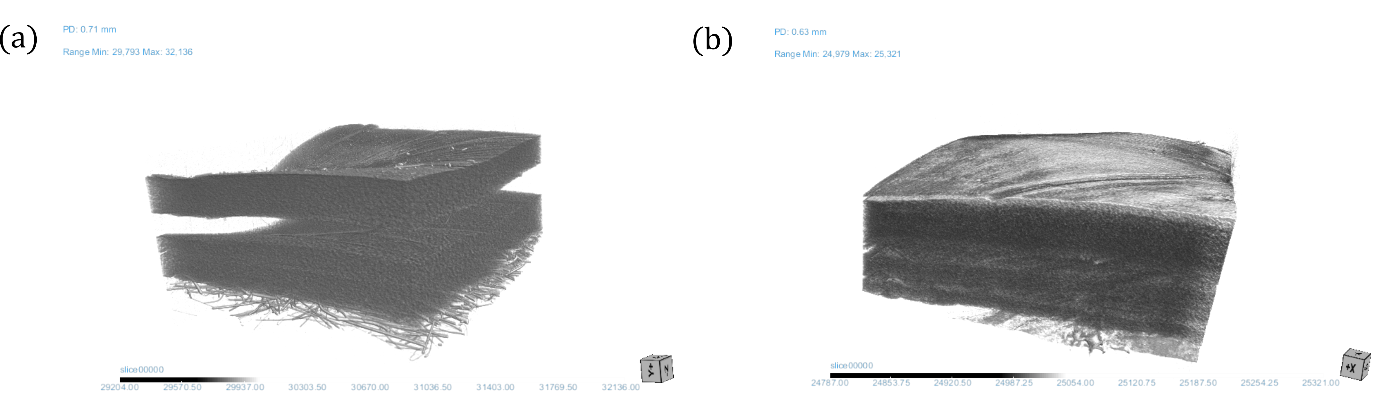
**Supplementary X-ray nano-CT**

**Figure S9.** Full tomograms taken on air electrodes discharged at (a) 0.1 mA cm^-2^ and (b) 1.5 mA cm^-2^


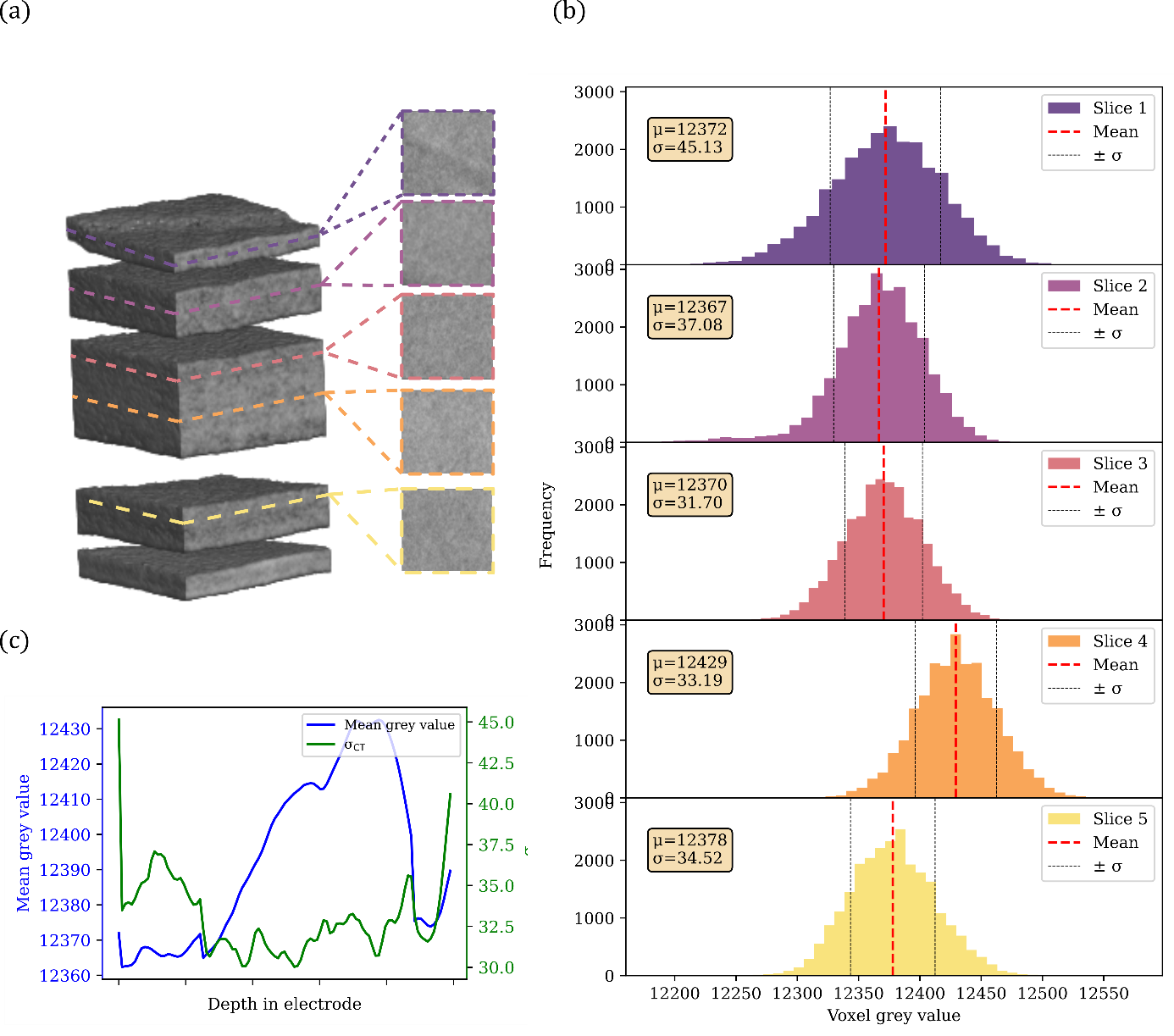


**Figure S10.** (a) X-ray nano-CT tomogram region of pristine air electrode with representative slices shown (b) histograms of pixel values for representative slices (c) Mean and standard deviation of CT intensity for all slices in pristine electrode vs depth in electrode

The pristine electrode was placed in a cell with electrolyte, rested without discharge, before removal, rinsing and drying in order to accurately replicate electrode conditions. Variation in the mean intensity indicates that there is some inhomogeneity in the density of the as-manufactured electrode material. The standard deviation of the voxel grey-values is largely constant, however, there are outliers at the edge of the electrode where some creasing may have occurred due to contact with the current collector mesh or separator.

**
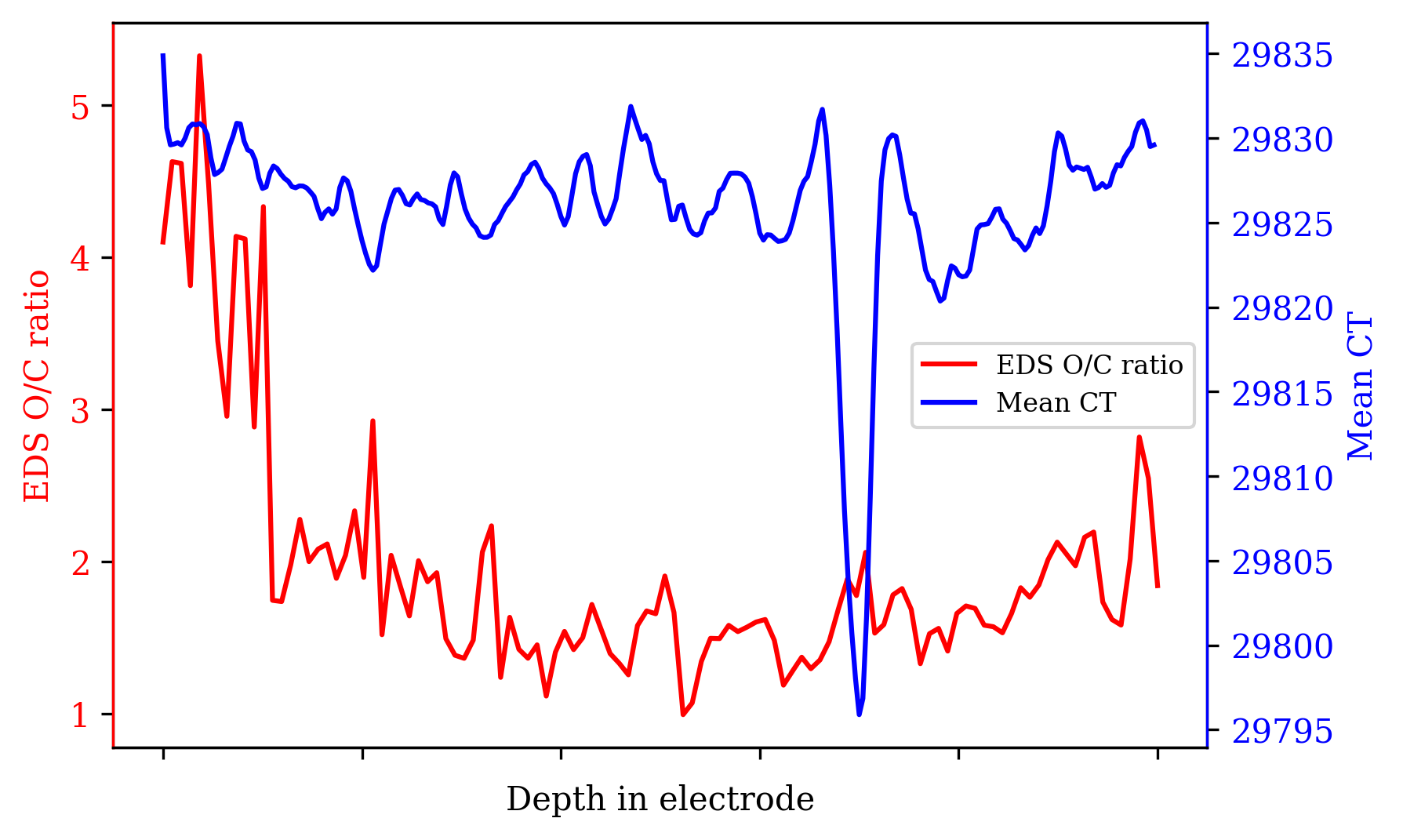
**

**Figure S11.** EDS O/C ratio through the depth of the electrode discharged at 0.1 mA cm^-2^ compared with the mean of the voxel grey level in each X-ray nano-CT slice through the depth of the electrode

Figure S10 compares the EDS O/C ratio measured through the depth of the electrode discharged at 0.1 mA cm^-2^ to the mean of the voxel grey level in each slice. There is no clear trend in the attenuation change, aside from one significant drop, potentially due to a large void in the electrode structure at this point.

**Sample preparation**


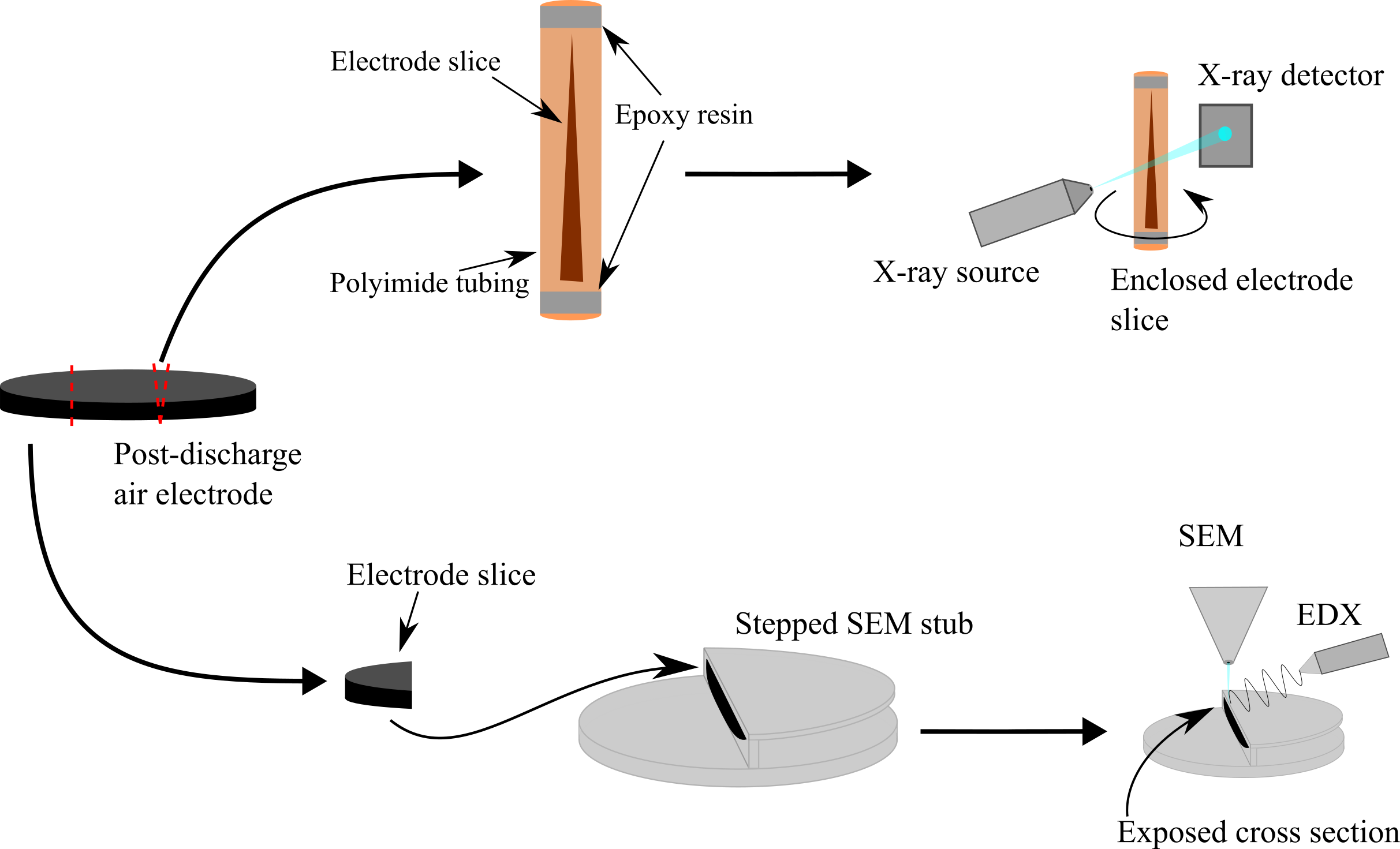


**Figure S12.** Schematic illustrating the sample preparation of discharged air electrodes for X-ray nano-CT (top) and SEM-EDS (bottom) characterisation

Sample preparation for X-ray nano-CT required the slicing of a thin, triangular slice and sealing it in a polyimide tube with epoxy resin. The purpose of the triangular slice is to have a region for measuring such that the thickness of the electrode, and thus X-ray attenuation, is relatively uniform at all orientations, allowing for improved reconstruction.

For SEM-EDS, the sample is sliced and the mounted on a stepped SEM stub such that the exposed cross-section is facing upwards. This allows for SEM-EDS characterisation of the cross-section.

**References**

[1] Sahapatsombut, U., Cheng, H., & Scott, K. (2013). Modelling the micro–macro homogeneous cycling behaviour of a lithium–air battery. *Journal of Power Sources*, *227*, 243–253. <https://doi.org/10.1016/j.jpowsour.2012.11.053>

[2] Sulzer, V., Marquis, S. G., Timms, R., Robinson, M., & Chapman, S. J. (2021). “Python Battery Mathematical Modelling (PyBaMM)”. *Journal of Open Research Software,9* (1), 14. <https://doi.org/10.5334/jors.309>
